# Empowering Movement: Enhancing Young Adults’ Physical Activity through Self-Determination Theory and Acceptance and Commitment Therapy-Based Intervention

**DOI:** 10.3390/bs14020130

**Published:** 2024-02-10

**Authors:** Dalit Lev-Arey, Tomer Gutman, Orr Levental

**Affiliations:** 1 Department of Psychology, The Academic College of Tel Aviv-Yaffo, Rabenu Yerucham, 2, Tel Avi Yaffo 6816811, Israel; tomergutman@gmail.com; 2 Department of Physical Education, Tel Hai Academic College, Upper Galilee 1220800, Israel; levental@gmail.com

**Keywords:** motivational dynamics, behavioral change, health promotion, psychological interventions, intervention efficacy, intrinsic motivation, exercise adherence

## Abstract

Objective: This study aims to investigate the effectiveness of a combined Self-Determination Theory (SDT) and Acceptance and Commitment Therapy (ACT) intervention, the ”Running Minds” program, in promoting physical activity (PA) among young adults. Methods: The intervention, consisting of eight sessions, targeted motivational and psychological barriers to PA. It intertwined SDT’s core components (autonomy, competence, relatedness) with ACT’s emphasis on mindfulness and value-driven actions. This study used a qualitative approach, collecting data through semi-structured interviews with twelve participants aged 20–35, conducted post-intervention. Results: Our reflexive thematic analysis of the interviews revealed five key themes: alignment with personal values, rewarding experience of the sessions, fulfillment of social connectedness, enhancement of both intrinsic and extrinsic motivation, and observable behavioral changes. These findings highlight the importance of aligning exercise with personal values and the role of supportive social environments in sustaining PA. Conclusions: The integration of SDT and ACT in the “Running Minds” program appears to be a viable approach for enhancing motivation and adherence to PA among young adults. This study offers valuable insights for future PA interventions, underscoring the need for strategies that consider psychological and social dimensions. Limitations and Future Research: Despite the promising results, limitations include potential recall bias and the short duration of the study. Further research, especially focusing on more diverse groups and employing longitudinal designs, is recommended to broaden and substantiate these findings.

## 1. Introduction

Physical activity (PA), widely recognized for its extensive health benefits, garners support across diverse disciplines, including medicine and psychology. Regular engagement in PA strengthens physical health by reducing risks that are associated with cardiovascular diseases, hypertension, and diabetes. Additionally, it enhances mental well-being, improving cognitive functions and managing issues like depression and anxiety [1,2,3]. PA also contributes to improved mood regulation, increased self-esteem, and enhanced cognition, collectively leading to a significantly better quality of life [4,5]. This positive impact extends to periods of increased stress, such as the COVID-19 pandemic, where regular PA was associated with maintained subjective well-being [6], further illustrating its broad and adaptable benefits. Yet despite this robust evidence, there is a disconcerting discrepancy: a substantial segment of the population does not achieve the PA levels that are recommended by health authorities [7,8]. The literature indicates a decline in PA during adolescence, often persisting into young adulthood, which underscores the need for intervention [9,10,11]. The issue of inactivity extends beyond personal choice, becoming a societal dilemma with consequences including spiraling healthcare costs and escalating public health concerns [12]. Physical inactivity is a formidable public health challenge, with the World Health Organization (WHO) attributing approximately 2 million deaths per year globally to inactivity, and with physical inactivity incurring significant economic costs [12]. Contributory factors include the sedentary nature of contemporary work and a general trend towards less active lifestyles [13].

Our study focuses on the 20–35-year-old demographic for several compelling reasons. WHO data show that 40% of this age group does not meet PA guidelines, an increase from 35% in 2018. This demographic is at a crucial stage for establishing lifelong PA habits, yet they face challenges such as career changes, marriage, and family responsibilities, which can limit their time for exercise [14,15]. Furthermore, this age bracket has been relatively neglected in PA research, which predominantly focuses on children and older adults [16]. Our study aims to address this gap, providing insights and strategies that are tailored to this crucial demographic.

Psychological interventions aimed at promoting PA have historically faced challenges, often due to overlooking the intricate relationship between motivation and behavior [17,18]. Self-Determination Theory (SDT) [19] provides insights into the motivational dynamics influencing PA. SDT emphasizes three key psychological needs—autonomy, relatedness, and competence—that are essential for motivating PA. Autonomy, or perceiving PA as self-chosen, is linked to sustained involvement and intrinsic pleasure [20]. Relatedness, a sense of connection, is nurtured through the social aspects of PA, like team sports [14,21]. Competence, feeling effective and skilled, is developed through personalized training and reaching fitness milestones, boosting confidence and persistence [22,23]. These components create a motivational ecosystem where autonomy empowers choice, relatedness fosters a supportive environment, and competence enhances confidence. Addressing these needs through SDT is crucial for overcoming barriers to PA and fostering a long-term commitment to an active lifestyle.

However, motivation alone does not dictate PA levels. To grapple with the challenges of low PA engagement and explore avenues for transforming motivation, we turn to Acceptance and Commitment Therapy (ACT). Situated within the Contextual Behavioral Science (CBS) paradigm [24,25], ACT highlights the significance of behavioral outcomes rather than their antecedents and integrates mindfulness practices into its approach. A recent meta-analysis conducted by Pears and Sutton [26] reviewed six ACT-based interventions for PA promotion, uncovering small to moderate beneficial effects.

SDT delves into the “why” behind behaviors, spotlighting autonomy, competence, and relatedness as critical for nurturing intrinsic motivation. In contrast, ACT focuses on the “how” by offering strategies like cognitive diffusion and acceptance to assist individuals in pursuing actions that are aligned with their values, even in the presence of psychological obstacles [25]. ACT helps individuals commit to PA as a value-driven pursuit, while SDT ensures that this commitment is fueled by self-motivation. This interplay between the “how” and “why” of behavior change is hypothesized to yield a more enduring transformation and represents a central theme of this study.

Drawing from the synergy of ACT and SDT, we have formulated an intervention targeting the complex nexus of motivation and behaviors in PA promotion. Appendix A presents an eight-module protocol encapsulating the core concepts that are imperative for effective PA engagement, titled “Running Minds”. The preliminary results from a pilot study with 94 sedentary college students lend substantial support to the efficacy of this integrated methodology [27], indicating the intervention’s considerable promise in enhancing personal motivation and activity levels—a promising foundation for further empirical inquiry and practical implementation in PA promotion.

The primary aim of this research was to investigate the effects of combining Self-Determination Theory (SDT) and Acceptance and Commitment Therapy (ACT) in a physical activity (PA) intervention, with a specific focus on the 20–35-year-old demographic. This age group was chosen due to compelling WHO data showing that 40% do not meet PA guidelines, a figure that has risen from 35% in 2018, indicating a growing need for effective interventions in this segment. While existing quantitative research, such as that by Lev Arey et al. [28], has established an initial understanding, it does not comprehensively address the full spectrum of psychological and behavioral factors influencing PA adherence. Our qualitative study aims to fill this gap, delving into the personal experiences of currently inactive young adults. By examining these experiences in depth, we seek to enrich our understanding of the factors that drive PA adherence and develop more effective strategies to promote PA among this crucial demographic.

## 2. Materials and Methods

### 2.1. Design

This study was a qualitative study, conducted with a sample of participants in a pilot trial design to assess the effectiveness of an SDT-ACT-based program, administered between July and September 2022. Data were collected through audio-taped semi-structured interviews with the participants who attended the PA enhancement intervention program.

### 2.2. Ethics

Prior to participation, individuals were thoroughly briefed on the study’s aims, methodologies, and ethical considerations, which included aspects of anonymity, confidentiality, and voluntary participation. Study procedures were approved and monitored by the Academic College of Tel Aviv-Yaffo Institutional Review Board (protocol number 2019158/02).

### 2.3. The Intervention

The intervention, titled “Running Minds”, as detailed in Lev Arey et al. [28], and further outlined in Appendix A, was designed to enhance self-compassion, understanding of cognitive traps, and promote behaviors that are congruent with personal values. This eight-session program, held on a college campus in Tel Aviv, chosen for its convenient access to public transportation, accommodated 10 to 12 participants per session, each lasting 90 min. The sessions, led by TG, an experienced sports psychologist and key developer of the program, were structured to deliver theory-based content. Educational materials and activities focused on topics such as personal values, behaviors aligned with these values, cognitive traps, and self-compassion, were utilized to ensure practical application of these concepts. Emphasizing experiential learning, the program encouraged participants to share personal experiences and engage in discussions with peers and professional staff, fostering a supportive learning environment. To enhance relatedness and peer support, participants were grouped into smaller units of 2–3 based on shared demographics like age and gender, promoting interaction and support outside of the formal sessions. The intervention’s fidelity and quality were planned to be assessed through specific measures, although details of these assessments and any modifications to the program, if made during the study, are not provided in this summary.

### 2.4. Sampling and Recruitment

After receiving ethical clearance from the institutional research review board, a multi-pronged recruitment strategy was implemented, including online advertisements, notices at community centers, and referrals from participants of a prior study. Our purposive sampling targeted young adults aged 20 to 35, predominantly leading sedentary lifestyles but showing a willingness to enhance their PA levels. The inclusion criteria required participants to be within the specified age bracket, have a health status that is conducive to physical exercise, and have a history of at least one year of physical inactivity, defined as not engaging in a minimum of 30 min of moderate-intensity PA on most days, per WHO 2022 guidelines. This deliberate sampling approach incorporated a broad spectrum of perspectives from individuals not meeting WHO’s PA standards. A demographic breakdown of our sample is detailed in Table 1, including age, gender, and relationship status, among other variables. Additionally, we considered the potential impact on the study results of pre-existing social connections among participants, which might arise from our recruitment methods.

### 2.5. Interview Process

Post-program, participants were approached for interviews within a two-week period to ensure the recency of their experiences. The semi-structured interviews were designed to balance methodological rigor with the necessary flexibility for an in-depth exploration of personal experiences. The interview questions, developed and refined collaboratively by the research team, underwent a phase of pre-testing to enhance clarity. These were based on existing research grounded in SDT and ACT principles. For sample interview questions, please refer to Table 2.

Conducted by a graduate psychology research assistant who was experienced in this methodology and knowledgeable about the intervention, the interviews aimed to foster an open and relatable dialogue. This effectiveness was partly due to the research assistant’s age proximity to the participants. Discussions began with topics of daily life and PA habits. They then delved into motivations for joining the program, the intervention’s impactful aspects, and participants’ detailed narratives of their experiences, all aided by strategic follow-up questions. To assess the immediate impact of the program, the interviews also inquired about participants’ ongoing PA practices in the two weeks following the program’s conclusion. The interviews, lasting between forty-five minutes and an hour and fifteen minutes with an average duration of 57 min, were conducted in Hebrew. They were subsequently translated by DL, a bilingual author, for this publication.

### 2.6. Data Analysis

Our research is embedded within a constructionist framework, which permits an in-depth exploration of the complex interplay between individual experiences, societal norms, and systemic factors that shape PA behaviors [28,29]. This approach is particularly relevant given our focus on young adults, who are experiencing significant life transitions and facing unique challenges that may influence their engagement in PA [15]. Building on insights from a preliminary pilot study [28], we have chosen to employ a qualitative approach to bridge identified gaps in existing research. The use of semi-structured interviews, which is consistent with our constructionist perspective, is intended to enrich the dataset and provide a comprehensive understanding of PA engagement among young adults. This methodological strategy is expected to contribute substantially to the development of nuanced PA enhancement strategies that align with our research aims and effectively address our core research questions.

The collected data underwent reflexive thematic analysis, adhering to the guidelines set by Braun and Clarke [27]. In the initial stage, the interview transcripts were independently read by the authors, DL and TG, both of whom are proficient in the theoretical frameworks that are relevant to this study. This step involved identifying subthemes such as “gender rigidity”, “motives for activity”, and “gender differences”. These subthemes resonated with complex narratives around autonomy, competence, and relatedness, as well as psychological flexibility and value-based action—central elements in our interpretive analysis within the constructionist paradigm [28,29]. The subthemes were then grouped into five main themes. A comparative review of these themes in line with inter-coder reliability guidelines [30] revealed no significant disagreements. To further validate these findings, a third researcher, OL, conducted an independent analysis, which yielded a Cohen’s kappa coefficient of 0.74, indicating a reasonably high level of agreement [31].

In recognition of our backgrounds in sport and exercise psychology, we have taken careful measures to mitigate potential biases through reflexive practices, triangulation of analysis, and consultation with non-specialist experts, ensuring a more balanced and objective interpretation of the data.

### 2.7. Ensuring Methodological Quality and Rigor

To ensure methodological rigor, our study employed a multifaceted validation approach, aligning with established academic standards. This approach included adopting a relativist orientation, which acknowledges the subjective nature of knowledge, and engaging in reflective practice, both of which served to enhance the study’s credibility. A key component of our strategy was member reflections, differing from traditional member checking. We encouraged participants to actively discuss the research findings, aiming to deepen mutual understanding and foster dialogue. Additionally, iterative questioning was pivotal during the interview process. This method enabled us to refine and expand our lines of inquiry based on participant feedback, leading to a more comprehensive understanding of the subject matter. This dynamic and responsive approach to data collection underscored the study’s adaptability and thoroughness. It allowed us to closely align our research with the intricate dynamics of interaction and perception that exist between participants and researchers.

## 3. Findings

Thirteen young adults—six females and seven males, aged 20 to 35—participated in “Running Minds”, an eight-session SDT-ACT intervention program. Twelve participants were interviewed within two weeks after the program concluded; one participant was unable to attend the interview. The data analysis revealed five primary themes: “Alignment of Participation with Internal Conditions”, “Program Sessions as Rewarding Events”, “Fulfilling the Need for Relatedness”, “Enhancement of Intrinsic and Extrinsic Motivation”, and “Behavior Change”. For a breakdown of these themes and subthemes, see Table 3.

### 3.1. Alignment of Participation with Appropriate Internal Conditions

The opportunity to participate in the program was met with enthusiasm by the participants. Once registered and attended, participants demonstrated their willingness to invest the required time and effort. This readiness was characterized by three subthemes that emerged from the data.

#### 3.1.1. The Readiness for Self-Exploration and the Desire to Make a Change

Participants exhibited a willingness to engage in self-exploration and a clear desire for personal growth. Participant #2 articulated this feeling, stating that “The invitation to participate was also a call for introspection and a commitment to personal growth. For me, it was a chance to delve into underlying issues and give the process an opportunity”.

Participant #1 echoed a similar experience, beginning with uncertainty and evolving into decisive action: “I realized that a change was necessary, and the program seemed tailor-made for my situation”. For these individuals, enrolling in the program was evidently a significant step towards embracing change.

#### 3.1.2. Challenges in Maintaining Consistent Exercise Habits

There was a significant discrepancy between the participants’ desired exercise habits and their actual levels of activity. Participant #7 disclosed that “I want to learn how to motivate myself... since moving to a new city, I haven’t managed to maintain a focus on my exercise routine”. Additionally, sustaining long-term exercise adherence posed a challenge for many.

Participant #9 candidly shared that “I’ve always started exercising and then stopped; I thought this time I could stick with it”.

#### 3.1.3. The Hope of Enhancing the Quantity and Quality of Exercise through the Program

Enrolling in the program was motivated by the anticipation of effectively increasing weekly exercise and maintaining it over the long term, thus narrowing the gap between participants’ ideal and actual exercise routines. All participants expressed dissatisfaction with their current level of PA, and the program offered a beacon of hope, bolstering their belief in the possibility of change. Participant #5 voiced her goal to acquire actionable strategies, remarking that “I registered with the hope of gaining tools that would aid in bolstering my perseverance”.

Participant #11 saw the program as timely, considering her recent uptick in PA: “I had started to engage in more rigorous sports just before this study. It presented a perfect chance to find a supportive structure that could help me maintain my newfound consistency”.

### 3.2. Program Meetings as Rewarding Events

The program’s meetings received positive evaluations, underscoring their role in sustaining an effective meeting environment. This reflects the notion of exercise as an appetitive behavior, which produces immediate positive outcomes, a quality that was similarly attributed to the meetings. Within this context, participants identified three significant subthemes that defined their experiences.

#### 3.2.1. Acquisition of New and Relevant Knowledge

The program’s psychoeducational component significantly enhanced participant engagement, offering content that was both relevant and practically applicable. This positive aspect is underscored by Participant #12’s feedback that “The tools we discussed were mostly important and applicable to me”, indicating the practical utility of the presented material. However, a notable limitation emerged in the transition from educational engagement to behavioral change.

Participant #10’s perspective sheds light on this challenge: “Although the discussions about values and acceptance were intriguing, they failed to provide the impetus I needed to spring into action”. This statement highlights a critical gap between the intellectual appreciation of the content and its effectiveness in catalyzing actual behavioral shifts among participants.

In essence, while the psychoeducational elements of the program were successful in engaging and resonating with the participants, their efficacy in driving significant behavioral changes was variable, pointing to the need for strategies that better facilitate the application of learned concepts into practical actions.

#### 3.2.2. Facilitating Conditions (Time, Duration, Location, Partners)

The intervention was held on a college campus, offering easy access for most participants. Its timing after work hours and during the summer was a key factor in its positive reception. Participant #4, living nearby, appreciated this convenience: “Since I live nearby, I decided to dedicate my evenings to the program”. However, for some, attending posed challenges due to distance or other commitments. Enrolling with friends or life partners emerged as a motivational factor, enhancing commitment.

Participant #3’s experience reflects this: “We both were keen to participate, so I joined the research with my partner. We were serious about it and coordinated our schedules”. This indicates that while pre-existing relationships generally motivated participation, they also brought complexities, which merit further discussion.

#### 3.2.3. Insight Gained through Self-Work

The intervention centered around self-reflection and various contemplative practices, focusing on introspection. Participants recognized the value of these exercises in personal development. Participant #8’s experience is telling: “The most profound insights I gained were related to my beliefs and thoughts about myself, my approach to physical activity, and the effort it requires from me”. This suggests a deep personal impact from the exercises.

Participant #10 also underscored the effectiveness of the structured exercises: “The tasks that offered me the most personal support were those we defined and discussed collectively, filling out a worksheet, then sharing and exploring practical applications”. His comments highlight the practical benefits of these activities, emphasizing their role in facilitating personal growth and application.

### 3.3. Fulfilling the Need for Relatedness

The intervention was designed to create a secure environment, foster mutual dedication, and nurture authenticity. Within this framework, three subthemes emerged from the interviews.

#### 3.3.1. Establishing a Connection with the Instructor

Participants consistently praised the instructor for his supportive and insightful approach, which they felt was genuinely aimed at positively impacting their lives. Participant #5 shared her thoughts on this: “He enlightened us with knowledge and fresh perspectives. His display of authenticity and transparency fostered a nonjudgmental ambiance throughout the meetings”. This comment highlights how the instructor’s genuine and open manner contributed to creating a comfortable and accepting environment in the group sessions.

Participant #6 echoed participant #5’s sentiments, further emphasizing the positive group dynamic: “The group dynamic was excellent; everyone was amiable, and the instructor established a real connection with us, emanating sincerity and genuineness”. Her experience reflects the instructor’s ability to not only connect with the participants on a personal level but also to cultivate a friendly and sincere atmosphere within the group, enhancing the overall experience.

#### 3.3.2. Sharing Difficulties and the Experience of Common Humanity

The group sessions, led by the instructor, were designed to create a safe and open environment. This setup encouraged participants to discuss their personal obstacles, challenges, and past hardships. The dynamics of the group made it easier for individuals to share experiences that they might usually keep private. Participant #7 provided a notable example of this effect. He is usually a very private person, not inclined to share his personal issues. But in the group, he experienced a significant change: “I am generally a private individual, habitually guarding my personal struggles. Sharing isn’t in my nature. However, in this setting, I encountered something truly transformative—a sense of camaraderie emerged as we all discussed our similar challenges. This fostered an atmosphere of acceptance, reassuring me that it’s okay to open up about my difficulties”. His experience underscores how the supportive group environment, fostered by the instructor, helped members feel comfortable enough to share their struggles.

However, the experience of sharing and engagement was not uniform across all participants. Participant #2 pointed out some limitations, particularly related to the size of the group: “The group was too large... I felt that those who spoke more were more noticeable or had a stronger personal connection with the instructor, so they might have gained more benefits”. Her observation suggests that in a larger group setting, not everyone felt equally heard or connected, which could impact the extent to which they shared and benefited from the session.

#### 3.3.3. Exploring the Dynamics of Social Connections within the Program

The program, aimed at fostering a sense of shared humanity, yielded mixed results in terms of facilitating lasting personal connections among participants. Although participants were organized into small teams of 2–3 participants, with the intention of promoting support between sessions, the effectiveness of this approach varied significantly. Collaboration within these teams also exhibited considerable variability, as highlighted by Participant #8, who observed that “The discussions during the sessions were fruitful, yet outside of those meetings, we hardly communicated”. Participant #4 echoed this sentiment, noting that “It functioned minimally, with little follow-up or meaningful communication among us”.

In contrast, the program had a notable positive impact on reinforcing pre-existing friendships. Participant #9, who attended the program with a friend, emphasized the benefits of this arrangement, stating that “Attending with a friend allowed for a shared journey towards healthier living. It was enriching to strengthen existing bonds and form new connections with others”. Interestingly, these relationships had a profound effect on participants, surpassing the influence of the educational content itself.

### 3.4. Enhancement of Intrinsic and Extrinsic Motivation

The analysis of the intervention reveals a notable enhancement in the participants’ motivation, as discerned from interviews and coding. This improvement is examined through two distinct subthemes, each delving into the origins and behavioral impacts of the motivational increase. These themes underscore the roles of personal values as enduring motivators and the influence of external motivation between sessions in sustaining engagement.

#### 3.4.1. Values as Long-Term Motivators

Central to the increase in intrinsic motivation were personal values. The intervention facilitated a deep engagement with participants’ intrinsic values, establishing a link between these values and the physical act of exercising. This awareness acted as a bridge, connecting exercise with foundational motivations. Participant #11 articulated the significance of this connection: “Understanding why I exercise and its connection to my values was crucial... being aligned with your values encourages persistence and enhances motivation”. This indicates that aligning activities with personal values can significantly bolster motivation.

Participant #7’s narrative presents a nuanced view of this dynamic. Describing himself as competitive, he found that this trait did not always lead to positive outcomes in PA. However, the intervention’s focus on values provided him with a transformative insight. He reflected that “The distinction between acting on values versus goals resonated with me deeply... It was refreshing to learn about leaning on values as an alternative source of motivation”. His experience underscores the impact of value-based motivation, especially when goal-oriented approaches fall short.

#### 3.4.2. Inter-Session External Motivation

The design of the program played a significant role in fostering external motivation, particularly through the structure of the sessions, which encouraged participants to share and discuss their activities on a weekly basis. Participant #11’s experience illuminates this effect, as she notes that “On weeks when I knew I would be sharing my experiences with the group, my commitment intensified”. This observation suggests that the anticipation of participating in these discussions served as a significant motivator, enhancing her engagement in the program.

Participant #2’s account echoes this sentiment. She found that the prospect of forthcoming group meetings spurred her into action, remarking that “The knowledge of an impending meeting would prompt me to do something worth sharing”. Her statement indicates that the scheduled sessions provided a compelling reason to stay active and involved, particularly during times when her personal motivation might have been waning.

However, it is important to recognize that this increase in motivation was not a universal experience among all participants. Some did not report experiencing the same level of motivational boost from the session structure, highlighting the variability in how individuals respond to external motivational factors in such interventions.

### 3.5. Behavior Change

The intervention’s primary objective was to instigate enduring and sustainable changes in participants’ behavior. Several subthemes emerged as being instrumental in achieving this objective.

#### 3.5.1. Appetitive Training Building

The provided guidance, aimed at fostering more pleasant and less strenuous exercise experiences, did indeed result in a notable change in the participants’ attitudes toward their training sessions. Participant #6 articulated this significant shift, saying that “The most important change for me has been learning not to push myself too hard. Discovering that brief spells of outdoor activity, as little as 10–20 min without pushing to fatigue, can be sufficient. This has allowed me to look forward to training again soon. It’s crucial that the activity feels rewarding and, ideally, pleasurable”.

Participant #1 also shared the behavioral modification that this realization fostered in him: “...particularly the insight that exercise shouldn’t be about perfectionism. There’s no need to aim to be the best athlete out there. Running should be for your own enjoyment, and merely participating in the activity is beneficial and feels rewarding... I run, and it brings me joy”.

#### 3.5.2. Enhancing PA in Daily Life Tasks

The reframing of everyday tasks as opportunities for incorporating PA prompted another significant behavioral change. Participants began to see routine actions, such as ascending stairs or walking to the store, as chances to increase their overall movement and improve their health. Participant #3 noted this transformation in his habits: “I’ve observed that on my walks with the dog, which can be quite lengthy, I sometimes just wear sandals. But then it struck me that I enjoy these walks and they’re good for my health. If I pick up the pace and raise my heart rate, sandals aren’t suitable. So, I’ve started to wear comfortable sneakers for our walks”. Participant #12 reflected on a sustained behavioral shift, adding that “There are behaviors that developed during the eight weeks that have persisted, like opting to walk instead of taking the bus, or choosing the stairs over the elevator. This change occurred within those weeks and has stayed with me; it’s become a part of my everyday life”.

#### 3.5.3. Strengthening Self-Regulation

From the interviews, it became evident that there was an enhancement in the participants’ self-regulatory abilities. They shared insights about becoming more mindful of their inner dialogue, which, in turn, enabled them to exercise greater control over their exercise routines. Participant #7, for instance, conveyed this heightened self-awareness: “During the interviews, I often mentioned how, at times, when I prepared to engage in a workout, there was a little voice that tempted me to stay on the couch, arguing that it would be more enjoyable. I learned through the interviews that recognizing this inner dialogue and then taking actions contrary to it was pivotal for maintaining a consistent exercise routine”.

However, it is important to note that despite these improvements, self-regulation did not uniformly advance for all participants. Life changes, such as starting a new job or relocating to a new apartment, often hindered the progress of some individuals in this aspect. Nonetheless, these participants consistently acknowledged the significance of self-regulation within the program, as revealed through their interview responses. Participant #4’s experience illustrates this, as he recalled a key message from the interviews: “When discussing my experiences during the interviews, I frequently mentioned the advice to initiate a simple action like rolling out a yoga mat or just tying your sneakers when lacking the energy to engage in physical activity. This principle, which I discussed in the interviews, extends to other areas of my life, especially when dealing with job changes and disruptions to my regular schedule”.

## 4. Discussion

In the current study, we embarked on an investigation into the effectiveness of an intervention rooted in the integration of two established psychological frameworks: ACT and SDT [28]. Our primary objective was to gain a deeper understanding of participants’ experiences during the program sessions and their impact on their engagement in PA and actual behaviors. To better understand the complex issues at play, we selected qualitative interviews as our principal method for data collection. This approach created an opportunity for participants to express their experiences and thoughts regarding the intervention. By focusing on qualitative research, we were able to grasp a variety of experiences and perceptions that might not be as apparent through quantitative methods. The outcomes of our analysis yielded five salient themes, each contributing to an enriched comprehension of the intervention’s efficacy. These themes, namely, “Alignment of Participation with Internal Conditions”, “Program Sessions as Rewarding Events”, “Fulfilling the Need for Relatedness”, “Enhancement of Intrinsic and Extrinsic Motivation”, and “Behavior Change”, collectively provide a multi-dimensional perspective on the integration of ACT and SDT in encouraging PA adherence. The results of this study found strong theoretical underpinnings in both SDT and ACT, each shedding light on different dimensions of participants’ experience.

### 4.1. Self-Determination Theory (SDT)

SDT posits that individuals have three basic psychological needs—competence, autonomy, and relatedness—that facilitate optimal development and function [32]. In the present study, the theme of “Fulfilling the Need for Relatedness” dovetails precisely with SDT’s construct of relatedness. Participants not only connected with the instructor but also felt a sense of common humanity with their peers, fulfilling their need for relatedness and thereby enhancing their motivation to engage in PA. Furthermore, the theme “Enhancement of Intrinsic and Extrinsic Motivation” reflects SDT’s dual emphasis on types of motivation. Our study’s participants cited both internal drivers, such as personal values, and external factors, like the structure of the sessions, as contributing to their motivation. This aligns with SDT’s distinction between intrinsic and extrinsic motivation and underscores the importance of satisfying multiple types of motivational drives in behavioral change programs. Specifically, by emphasizing personal values, the intervention potentially increases participants’ autonomy levels in the context of PA.

### 4.2. Acceptance and Commitment Therapy (ACT)

ACT, with its emphasis on acceptance, mindfulness, and value-based action, offers another valuable perspective for interpreting this study’s findings [33]. The theme of “Alignment of Participation with Internal Conditions” encapsulates the context that fosters a willingness to change, reflecting ACT’s principle of acceptance. Participants recognized their internal challenges and committed to addressing them, thus embodying ACT’s core concept of confronting difficulties while acting in accordance with values. This commitment signifies a behavioral change that extends beyond PA; it includes a transformation in how participants navigate internal barriers, such as negative self-talk or emotional discomfort. This aligns with ACT’s goal of promoting behavioral flexibility in service of valued living. The theme of “Enhancement of Intrinsic and Extrinsic Motivation” also aligns with ACT, particularly in the subtheme of “Values as Long-term Drivers”. Participants who linked their PA to deeper values found a strong form of internal motivation, which is consistent with ACT’s focus on committed action based on values. This theme is in line with the behavioral foundations of ACT, which emphasize values as chosen qualities of purposeful action.

### 4.3. Comparison with Existing Research

Our study’s findings both validate and extend previous research in significant ways. A central theme that we identified, “Enhancement of Intrinsic and Extrinsic Motivation”, aligns with earlier studies, confirming the critical role of motivation in encouraging PA and facilitating behavior change [34,35,36]. However, our research goes a step further by delving into the roots of this motivation. Beyond previous studies, we explore deeper sources of motivation, including personal values and expectations, offering a nuanced view of how these factors drive sustained engagement in PA programs.

The theme of “Fulfilling the Need for Relatedness” closely aligns with the concept of relatedness within SDT. Previous research [22,37,38] has firmly established the critical role of social support in PA adherence. Our study adds to this body of knowledge by shedding further light on this aspect. We found that participants in our intervention program derived greater satisfaction from sharing their personal journeys within the group than from assistance and communication between sessions. This suggests that the act of sharing personal experiences related to PA may have a more profound impact on fulfilling the need for relatedness than external interactions or support. Furthermore, our research explores the multifaceted ways in which relatedness can be nurtured. We also emphasize the pivotal roles that are played by the instructor and group dynamics in cultivating a sense of relatedness among participants. These insights offer valuable practical implications for the development of future programs aimed at promoting relatedness in the context of PA, underscoring the importance of creating an environment where individuals can share their experiences and build meaningful connections within a group setting.

Furthermore, the concept of “Program Sessions as Rewarding Events” introduces a new perspective by emphasizing the role of rewarding experiences in fostering sustained behavior change. Our study specifically focuses on the elements of program sessions that participants found satisfying, such as acquiring useful knowledge and receiving communal support from the group. This aspect of our research contributes to a broader understanding of effective strategies for initiating and maintaining behavior change in relation to PA. Additionally, the overarching theme of “Behavior Change” encompasses subthemes like “Appetitive Training Building” and “Strengthening Self-Regulation”. These subthemes explore various methods for making behavioral shifts more enduring. Another subtheme, “Enhancing PA in Daily Tasks”, proposes a practical approach to integrating PA into everyday life. While much research centers on structured exercise programs, our findings suggest that incorporating incidental physical activity into daily routines can significantly increase overall PA levels.

### 4.4. Explanations for Emergent Results

A notable aspect of our research is the demonstrated impact of logistical factors on participant engagement. Although traditional research often emphasizes intrinsic factors like individual motivation or readiness for behavior change, it tends to overlook extrinsic, operational elements such as timing and location [39]. Our findings indicate that these factors are far from trivial; in fact, convenient timing and accessible locations acted as critical facilitators, mitigating barriers to entry and participation. These elements served as a “soft infrastructure” that streamlined the engagement process.

Moreover, the instructor’s pivotal role in “Fulfilling the Need for Relatedness” provides a compelling narrative for the program’s success beyond mere content delivery. The nature of the interaction between the instructor and participants emerged as a significant contributor to the program’s efficacy. Participants perceived the instructor’s accessibility, expertise, and genuine concern for their well-being as cornerstones of an engaging and impactful learning experience. This strong rapport likely bolstered participants’ sense of connectedness, engagement, and motivation, potentially creating a psychologically secure environment that was conducive to open discussions of challenges and an affirmative response to the program’s objectives. This dynamic aligns with the actionable insights that are prescribed by both ACT and SDT [40,41]. The dynamics between the instructor and participants may highlight the importance of the broader social context in behavior change. This suggests that the instructor’s role transcends being merely an informant and becomes that of a catalyst for transformation [22].

### 4.5. Limitations and Future Research

While we took measures to ensure methodological rigor, our study is not without limitations that may impact the interpretation of our findings. The primary data collection through retrospective interviews, conducted within two weeks post-program, provided depth but also carried the risk of recall bias due to the reliance on participant memory and self-reporting. Real-time fluctuations in motivation and psychological flexibility, which are inherently dynamic, may not have been fully captured by such interviews. Another limitation is the study’s short duration, which provides initial insights but does not permit observation of long-term behavior changes or intervention sustainability. Additionally, the potential cultural bias, due to the sample being predominantly educated young adults from central Israel, may limit the generalizability of our findings, as cultural nuances can significantly influence behaviors and perceptions [42].

To address these gaps, future research should consider expanding the demographic diversity, potentially examining interventions among adolescents or older adults to enhance generalizability. Employing real-time data collection methods, such as participant diaries or ecological momentary assessment, could offer more immediate insights into participants’ experiences. A longitudinal design could examine the durability of behavior changes over time, contributing to our understanding of the intervention’s long-term impact. Incorporating a mixed-methods approach with qualitative feedback and quantitative data, perhaps via wearable technology and physiological measures, could provide a more comprehensive evaluation of the intervention’s effects. Our findings, while instructive, should be viewed critically when considering their application to other contexts or populations, and they should act as a springboard for further research rather than definitive conclusions [43,44]. Caution is advised in generalizing our results, which were derived from a specific group. The current research did not include data on the behavioral changes that were experienced by each participant. These could be explored in future research based on the current findings and conclusions. Moreover, our study, guided primarily by SDT and ACT, provided focused insights into factors influencing physical activity adherence. However, it is noteworthy that this specific theoretical orientation may not have captured all aspects of the participants’ experiences. While this approach was integral to our research objectives, it is possible that responses or experiences not aligning directly with SDT and ACT principles were not fully explored, suggesting scope for further research to uncover other psychological or environmental influences on physical activity.

### 4.6. Implications

While acknowledging the limitations of our study, it represents a significant step forward in integrating SDT with ACT to enhance PA. Grounded in our constructivist worldview, this research provides a foundation for future explorations that combine various psychological theories to develop more effective intervention methods. From a practical standpoint, our findings offer valuable insights for policy makers and healthcare professionals. These insights can inform the development of strategies aimed at reducing sedentary behaviors and improving public health, aligning with the guidelines that are set forth by the WHO. Our research highlights the importance of policy initiatives and further research that align with our philosophical perspectives on knowledge and reality. It emphasizes the need for a diverse and multifaceted approach to address sedentary lifestyles and promote overall well-being.

## 5. Conclusions

This study demonstrates the effectiveness of integrating SDT and ACT in enhancing PA among young adults. The intervention, “Running Minds”, effectively increased intrinsic and extrinsic motivation, fostered a sense of relatedness, and encouraged behavior change in terms of PA. Participants reported an increased commitment to PA, highlighting the importance of supportive environments and the role of personal values in sustaining activity levels. While the study faced limitations such as potential recall bias and cultural bias, it paves the way for future research, particularly in broadening demographic inclusivity and employing longitudinal study designs. This research contributes to the field of health promotion by offering insights into designing effective PA interventions for young adults. It underscores the need for multifaceted and tailored approaches in health behavior interventions, emphasizing the importance of psychological and social dimensions in promoting physical activity and overall well-being.

## Figures and Tables

**Table 1:** Demographic data of participants.

Participant	Gender	Age	Status	Pre-Existing Social Connections
Participant #1	M	34	Single	No
Participant #2	F	24	Married	Yes
Participant #3	M	25	Single	No
Participant #4	M	35	Married	No
Participant #5	F	27	Single	No
Participant #6	F	24	Single	Yes
Participant #7	M	31	In Relationship	No
Participant #8	M	31	Single	No
Participant #9	F	27	In Relationship	Yes
Participant #10	M	24	Single	No
Participant #11	F	35	Married	No
Participant #12	F	26	In Relationship	Yes

**Table 2:** Example interview questions.

**1**	Why did you decide to register for the program?
**2**	How were the contents and exercises? What was more and less valuable?
**3**	How was the interaction with the instructor? With peers?
**4**	How did participation impact your physical activity and exercise?
**5**	Can you discuss which program elements you have applied?
**6**	What are the main difficulties you are experiencing around exercising?
**7**	Have you noticed any changes in your behaviors?

**Table 3:** Primary themes and subthemes.

Theme	Subthemes
1. Alignment of participation with internal condition	- The readiness for self-exploration and the desire to make change - Challenges in maintaining consistent exercise habits - The hope of enhancing the quantity and quality of exercise through the program
2. Program meetings as rewarding events	- Acquisition of new and relevant knowledge - Facilitating conditions (time, duration, location) - Insight gained through self-work
3. Fulfilling the need for relatedness	- Establishing a connection with the instructor - Sharing difficulties - Experience of common humanity - The impact of existing relationships between participants while failing to establish new personal connections
4. Enhancement of intrinsic and extrinsic motivation	- Values as a long-term contingency - Inter-session external motivation
5. Behavior change	- Appetitive training building - Enhancing daily PA - Strengthening self-regulation

## Data Availability

The data presented in this study are available on request from the corresponding author. The data are not publicly available to avoid potential identification of interviewees based on comments made.

## Appendix A

**Table A1:** “Running Minds” Intervention Overview.

Module	Content	Description	Theory References
0	Registration	- Publication and Invitation: “Running Mind—a program for enjoyable and satisfied exercise”.	The need for Autonomy—free choice registration.
1	Introduction and Motivational Personal Investigation	- Representation of the program schedule and requirements. - Acquaintance. - Basic motivational intervention—pros and cons of the current and desired exercise status in participants’ lives. - Filtering non-ready participants by declaring an exit point between meeting 1 and 2.	The need for Autonomy—setting and personal connection enhancement.
2	Values and Value-Driven Behaviors	- Getting in emotional touch with participants’ deeper values that motivated them to exercise. - Exploration of their internal experience that “gets them on their way” to exercise.	Values and integrative motivation.
3	Thinking Traps	- Psychoeducation about the human mind and cognitive traps (non-adaptive interpretations about PA). - Personal exploration of each participant’s thinking traps.	The need for relatedness—common humanity (“I’m human like everybody else”).
4	Willingness	- Increasing participants’ ability to accept their uncomfortable internal experience when they are going towards what is important to them in PA.	The need for autonomy and competence: increase the ability to accept internal experience and to move towards values.
5	Applied Behavioral Analysis	- Psychoeducation: long- and short-term consequences of PA. - Small group discussions about each participant’s common behaviors.	Behavior analysis of my PA—How to make it more appetitive?
6	Self-Compassion	- Psychoeducation and exercises - Discussion about self-compassionate PA - Personal exploration of the implementation of self-compassion in each participant’s daily PA.	
7	Relatedness in Action	- Follow-up and self-examination about what are considered workable and non-workable behaviors in the current PA status. - Dividing into small support groups.	
8	Summary and Relapse Prevention	- Psychoeducation about relapse prevention - “What I have achieved” session and planning for the future.	

PA—physical activity.
